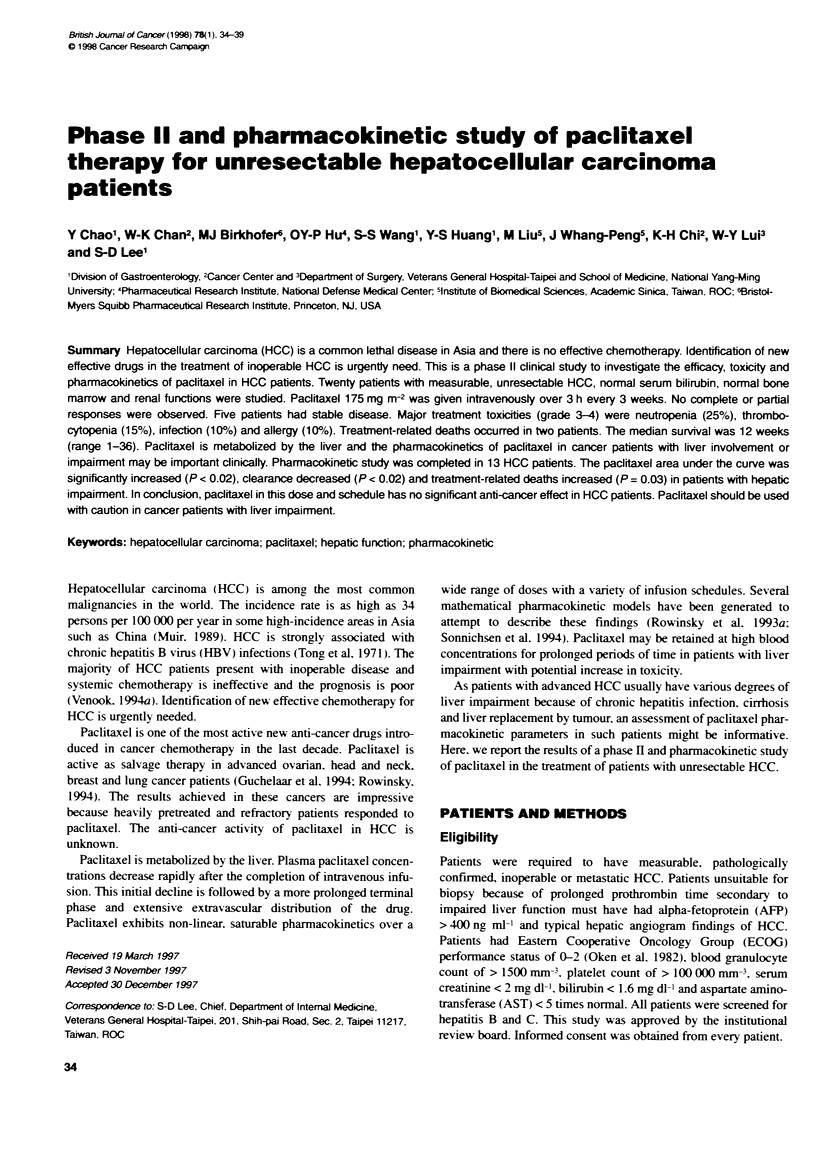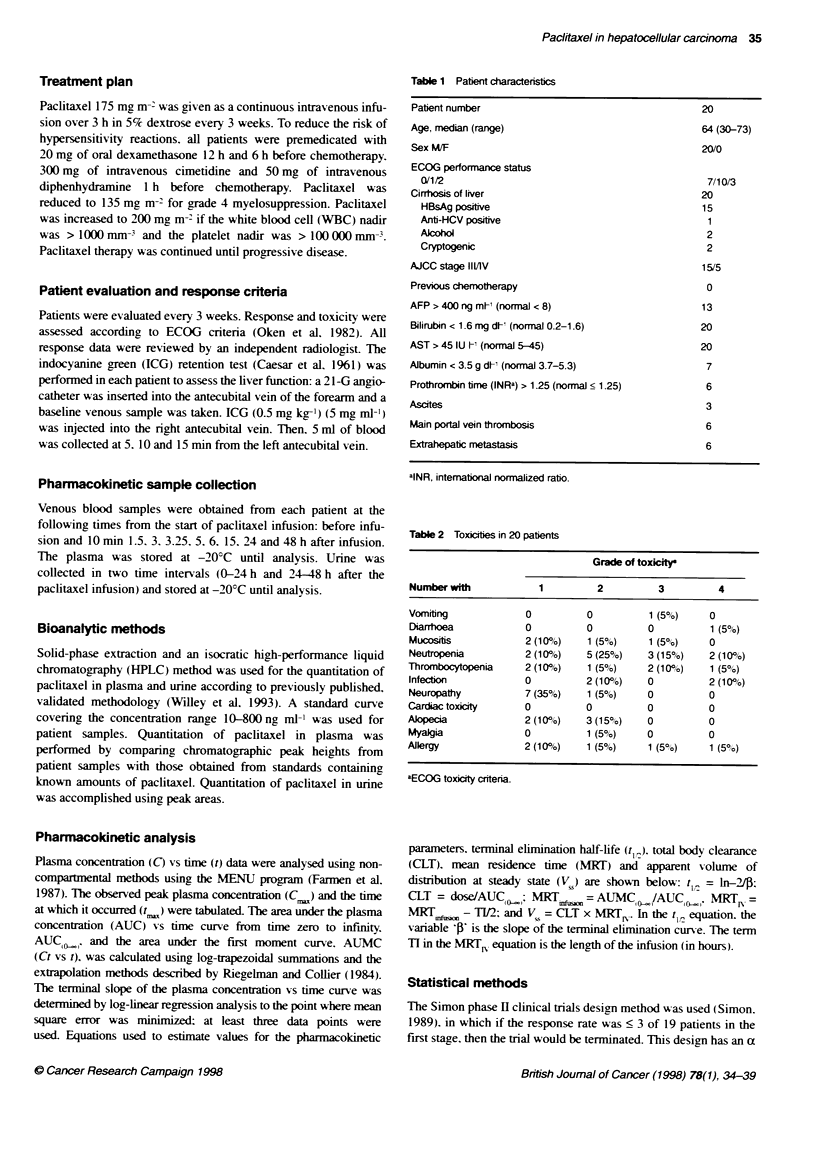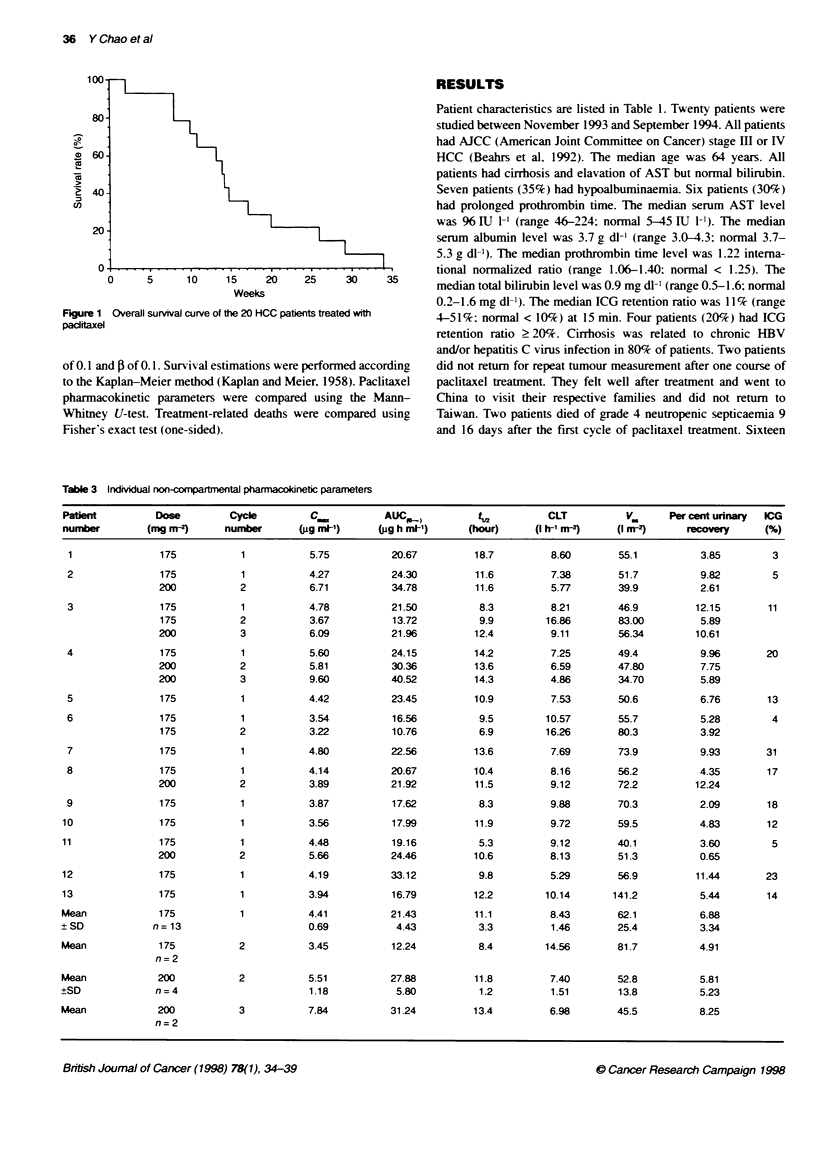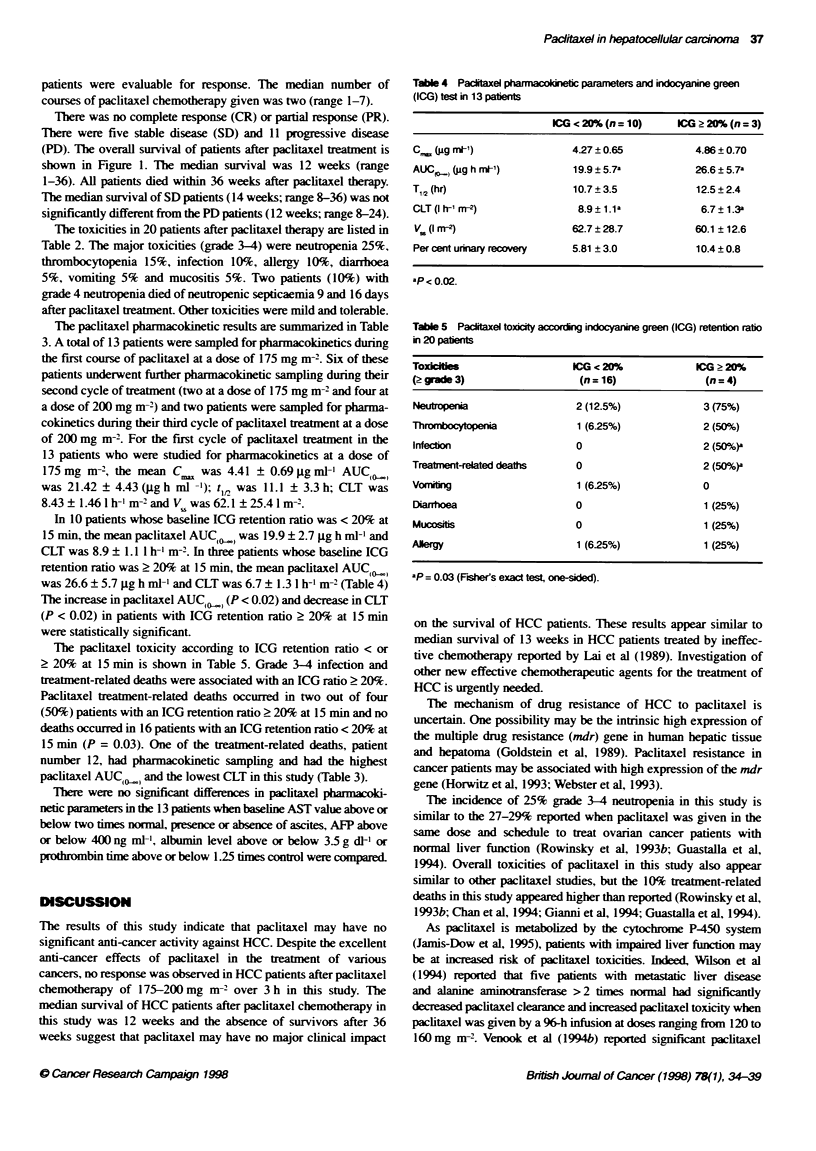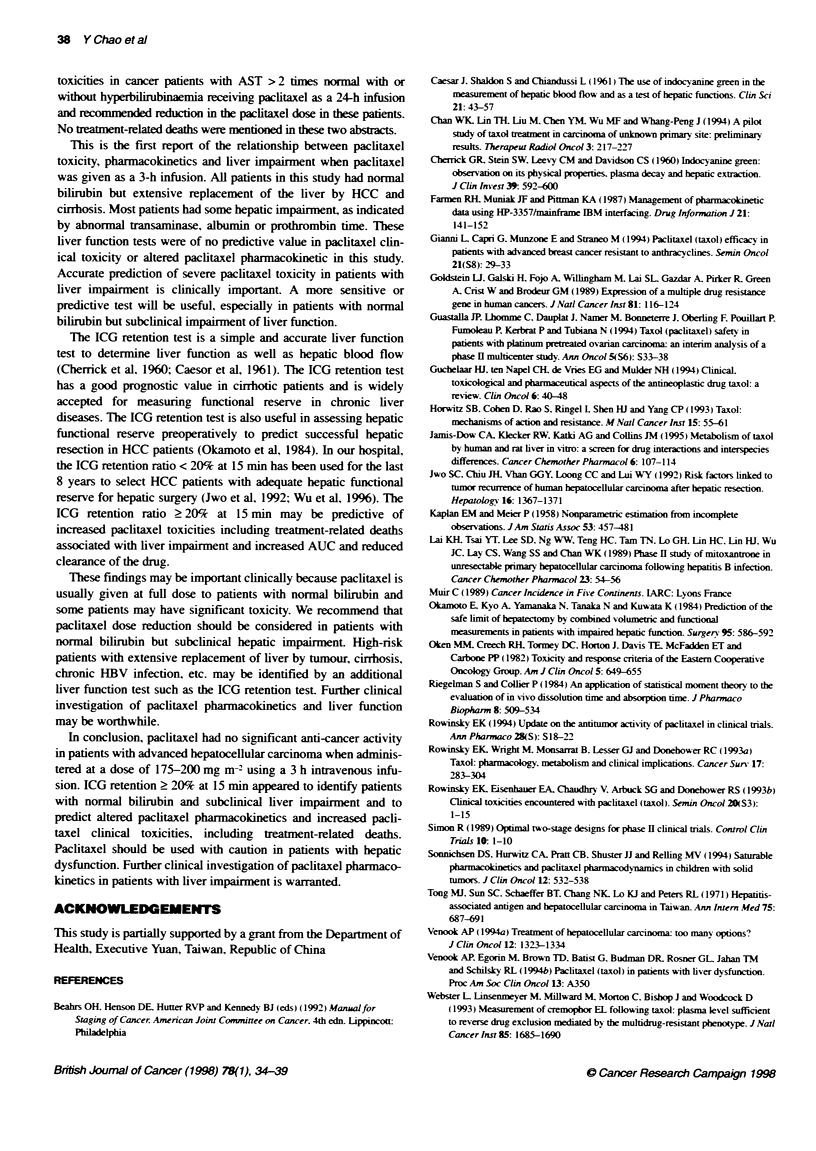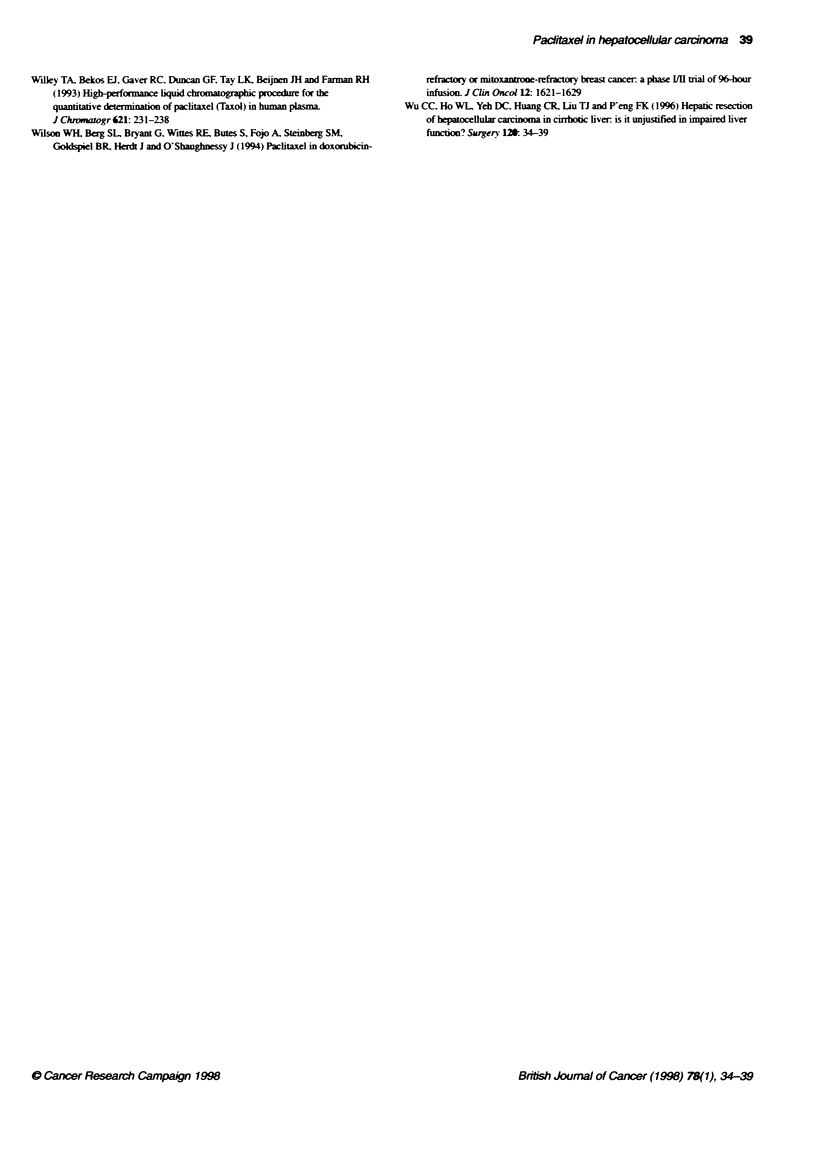# Phase II and pharmacokinetic study of paclitaxel therapy for unresectable hepatocellular carcinoma patients.

**DOI:** 10.1038/bjc.1998.438

**Published:** 1998-07

**Authors:** Y. Chao, W. K. Chan, M. J. Birkhofer, O. Y. Hu, S. S. Wang, Y. S. Huang, M. Liu, J. Whang-Peng, K. H. Chi, W. Y. Lui, S. D. Lee

**Affiliations:** Division of Gastroenterology, Veterans General Hospital-Taipei and School of Medicine, National Yang-Ming University, Taiwan, ROC.

## Abstract

Hepatocellular carcinoma (HCC) is a common lethal disease in Asia and there is no effective chemotherapy. Identification of new effective drugs in the treatment of inoperable HCC is urgently need. This is a phase II clinical study to investigate the efficacy, toxicity and pharmacokinetics of paclitaxel in HCC patients. Twenty patients with measurable, unresectable HCC, normal serum bilirubin, normal bone marrow and renal functions were studied. Paclitaxel 175 mg m(-2) was given intravenously over 3 h every 3 weeks. No complete or partial responses were observed. Five patients had stable disease. Major treatment toxicities (grade 3-4) were neutropenia (25%), thrombocytopenia (15%), infection (10%) and allergy (10%). Treatment-related deaths occurred in two patients. The median survival was 12 weeks (range 1-36). Paclitaxel is metabolized by the liver and the pharmacokinetics of paclitaxel in cancer patients with liver involvement or impairment may be important clinically. Pharmacokinetic study was completed in 13 HCC patients. The paclitaxel area under the curve was significantly increased (P < 0.02), clearance decreased (P < 0.02) and treatment-related deaths increased (P = 0.03) in patients with hepatic impairment. In conclusion, paclitaxel in this dose and schedule has no significant anti-cancer effect in HCC patients. Paclitaxel should be used with caution in cancer patients with liver impairment.


British Journal of Cancer (1998) 78(1), 34–39
© 1998 Cancer Research Campaign

### Phase II and pharmacokinetic study of paclitaxel therapy for unresectable hepatocellular carcinoma patients

Y Chao[1], W-K Chan[2], MJ Birkhofer[6], OY-P Hu[4], S-S Wang[1], Y-S Huang[1], M Liu[5], J Whang-Peng[5], K-H Chi[2], W-Y Lui[3] and S-D Lee[1]

[1]Division of Gastroenterology, [2]Cancer Center and [3]Department of Surgery, Veterans General Hospital-Taipei and School of Medicine, National Yang-Ming University; [4]Pharmaceutical Research Institute, National Defense Medical Center; [5]Institute of Biomedical Sciences, Academic Sinica, Taiwan, ROC; [6]Bristol-Myers Squibb Pharmaceutical Research Institute, Princeton, NJ, USA

**Summary** Hepatocellular carcinoma (HCC) is a common lethal disease in Asia and there is no effective chemotherapy. Identification of new effective drugs in the treatment of inoperable HCC is urgently need. This is a phase II clinical study to investigate the efficacy, toxicity and pharmacokinetics of paclitaxel in HCC patients. Twenty patients with measurable, unresectable HCC, normal serum bilirubin, normal bone marrow and renal functions were studied. Paclitaxel 175 mg $m^{-2}$ was given intravenously over 3 h every 3 weeks. No complete or partial responses were observed. Five patients had stable disease. Major treatment toxicities (grade 3–4) were neutropenia (25%), thrombocytopenia (15%), infection (10%) and allergy (10%). Treatment-related deaths occurred in two patients. The median survival was 12 weeks (range 1–36). Paclitaxel is metabolized by the liver and the pharmacokinetics of paclitaxel in cancer patients with liver involvement or impairment may be important clinically. Pharmacokinetic study was completed in 13 HCC patients. The paclitaxel area under the curve was significantly increased ($P < 0.02$), clearance decreased ($P < 0.02$) and treatment-related deaths increased ($P = 0.03$) in patients with hepatic impairment. In conclusion, paclitaxel in this dose and schedule has no significant anti-cancer effect in HCC patients. Paclitaxel should be used with caution in cancer patients with liver impairment.

**Keywords:** hepatocellular carcinoma; paclitaxel; hepatic function; pharmacokinetic

Hepatocellular carcinoma (HCC) is among the most common malignancies in the world. The incidence rate is as high as 34 persons per 100 000 per year in some high-incidence areas in Asia such as China (Muir, 1989). HCC is strongly associated with chronic hepatitis B virus (HBV) infections (Tong et al, 1971). The majority of HCC patients present with inoperable disease and systemic chemotherapy is ineffective and the prognosis is poor (Venook, 1994*a*). Identification of new effective chemotherapy for HCC is urgently needed.

Paclitaxel is one of the most active new anti-cancer drugs introduced in cancer chemotherapy in the last decade. Paclitaxel is active as salvage therapy in advanced ovarian, head and neck, breast and lung cancer patients (Guchelaar et al, 1994; Rowinsky, 1994). The results achieved in these cancers are impressive because heavily pretreated and refractory patients responded to paclitaxel. The anti-cancer activity of paclitaxel in HCC is unknown.

Paclitaxel is metabolized by the liver. Plasma paclitaxel concentrations decrease rapidly after the completion of intravenous infusion. This initial decline is followed by a more prolonged terminal phase and extensive extravascular distribution of the drug. Paclitaxel exhibits non-linear, saturable pharmacokinetics over a wide range of doses with a variety of infusion schedules. Several mathematical pharmacokinetic models have been generated to attempt to describe these findings (Rowinsky et al, 1993*a*; Sonnichsen et al, 1994). Paclitaxel may be retained at high blood concentrations for prolonged periods of time in patients with liver impairment with potential increase in toxicity.

As patients with advanced HCC usually have various degrees of liver impairment because of chronic hepatitis infection, cirrhosis and liver replacement by tumour, an assessment of paclitaxel pharmacokinetic parameters in such patients might be informative. Here, we report the results of a phase II and pharmacokinetic study of paclitaxel in the treatment of patients with unresectable HCC.

### PATIENTS AND METHODS

### Eligibility

Patients were required to have measurable, pathologically confirmed, inoperable or metastatic HCC. Patients unsuitable for biopsy because of prolonged prothrombin time secondary to impaired liver function must have had alpha-fetoprotein (AFP) > 400 ng $ml^{-1}$ and typical hepatic angiogram findings of HCC. Patients had Eastern Cooperative Oncology Group (ECOG) performance status of 0–2 (Oken et al, 1982), blood granulocyte count of > 1500 $mm^{-3}$, platelet count of > 100 000 $mm^{-3}$, serum creatinine < 2 mg $dl^{-1}$, bilirubin < 1.6 mg $dl^{-1}$ and aspartate aminotransferase (AST) < 5 times normal. All patients were screened for hepatitis B and C. This study was approved by the institutional review board. Informed consent was obtained from every patient.

*Received 19 March 1997*
*Revised 3 November 1997*
*Accepted 30 December 1997*

*Correspondence to:* S-D Lee, Chief, Department of Internal Medicine, Veterans General Hospital-Taipei, 201, Shih-pai Road, Sec. 2, Taipei 11217, Taiwan, ROC

### Treatment plan

Paclitaxel 175 mg $m^{-2}$ was given as a continuous intravenous infusion over 3 h in 5% dextrose every 3 weeks. To reduce the risk of hypersensitivity reactions, all patients were premedicated with 20 mg of oral dexamethasone 12 h and 6 h before chemotherapy, 300 mg of intravenous cimetidine and 50 mg of intravenous diphenhydramine 1 h before chemotherapy. Paclitaxel was reduced to 135 mg $m^{-2}$ for grade 4 myelosuppression. Paclitaxel was increased to 200 mg $m^{-2}$ if the white blood cell (WBC) nadir was > 1000 $mm^{-3}$ and the platelet nadir was > 100 000 $mm^{-3}$. Paclitaxel therapy was continued until progressive disease.

### Patient evaluation and response criteria

Patients were evaluated every 3 weeks. Response and toxicity were assessed according to ECOG criteria (Oken et al, 1982). All response data were reviewed by an independent radiologist. The indocyanine green (ICG) retention test (Caesar et al, 1961) was performed in each patient to assess the liver function: a 21-G angiocatheter was inserted into the antecubital vein of the forearm and a baseline venous sample was taken. ICG (0.5 mg $kg^{-1}$) (5 mg $ml^{-1}$) was injected into the right antecubital vein. Then, 5 ml of blood was collected at 5, 10 and 15 min from the left antecubital vein.

### Pharmacokinetic sample collection

Venous blood samples were obtained from each patient at the following times from the start of paclitaxel infusion: before infusion and 10 min 1.5, 3, 3.25, 5, 6, 15, 24 and 48 h after infusion. The plasma was stored at –20°C until analysis. Urine was collected in two time intervals (0–24 h and 24–48 h after the paclitaxel infusion) and stored at –20°C until analysis.

### Bioanalytic methods

Solid-phase extraction and an isocratic high-performance liquid chromatography (HPLC) method was used for the quantitation of paclitaxel in plasma and urine according to previously published, validated methodology (Willey et al, 1993). A standard curve covering the concentration range 10–800 ng $ml^{-1}$ was used for patient samples. Quantitation of paclitaxel in plasma was performed by comparing chromatographic peak heights from patient samples with those obtained from standards containing known amounts of paclitaxel. Quantitation of paclitaxel in urine was accomplished using peak areas.

### Pharmacokinetic analysis

Plasma concentration ($C$) vs time ($t$) data were analysed using non-compartmental methods using the MENU program (Farmen et al, 1987). The observed peak plasma concentration ($C_{max}$) and the time at which it occurred ($t_{max}$) were tabulated. The area under the plasma concentration (AUC) vs time curve from time zero to infinity, $AUC_{(0-\infty)}$, and the area under the first moment curve, AUMC ($Ct$ vs $t$), was calculated using log-trapezoidal summations and the extrapolation methods described by Riegelman and Collier (1984). The terminal slope of the plasma concentration vs time curve was determined by log-linear regression analysis to the point where mean square error was minimized; at least three data points were used. Equations used to estimate values for the pharmacokinetic parameters, terminal elimination half-life ($t_{1/2}$), total body clearance (CLT), mean residence time (MRT) and apparent volume of distribution at steady state ($V_{ss}$) are shown below: $t_{1/2}$ = ln–2/β; CLT = dose/$AUC_{(0-\infty)}$; $MRT_{infusion} = AUMC_{(0-\infty)}/AUC_{(0-\infty)}$, $MRT_{IV} = MRT_{infusion}$ – TI/2; and $V_{ss}$ = CLT × $MRT_{IV}$. In the $t_{1/2}$ equation, the variable 'β' is the slope of the terminal elimination curve. The term TI in the $MRT_{IV}$ equation is the length of the infusion (in hours).

### Statistical methods

The Simon phase II clinical trials design method was used (Simon, 1989), in which if the response rate was ≤ 3 of 19 patients in the first stage, then the trial would be terminated. This design has an α

**Table 1** Patient characteristics

| | |
|---|---|
| Patient number | 20 |
| Age, median (range) | 64 (30–73) |
| Sex M/F | 20/0 |
| ECOG performance status | |
| 0/1/2 | 7/10/3 |
| Cirrhosis of liver | 20 |
| HBsAg positive | 15 |
| Anti-HCV positive | 1 |
| Alcohol | 2 |
| Cryptogenic | 2 |
| AJCC stage III/IV | 15/5 |
| Previous chemotherapy | 0 |
| AFP > 400 ng $ml^{-1}$ (normal < 8) | 13 |
| Bilirubin < 1.6 mg $dl^{-1}$ (normal 0.2–1.6) | 20 |
| AST > 45 IU $l^{-1}$ (normal 5–45) | 20 |
| Albumin < 3.5 g $dl^{-1}$ (normal 3.7–5.3) | 7 |
| Prothrombin time (INR[a]) > 1.25 (normal ≤ 1.25) | 6 |
| Ascites | 3 |
| Main portal vein thrombosis | 6 |
| Extrahepatic metastasis | 6 |

[a]INR, international normalized ratio.

**Table 2** Toxicities in 20 patients

| | Grade of toxicity[a] | | | |
|---|---|---|---|---|
| Number with | 1 | 2 | 3 | 4 |
| Vomiting | 0 | 0 | 1 (5%) | 0 |
| Diarrhoea | 0 | 0 | 0 | 1 (5%) |
| Mucositis | 2 (10%) | 1 (5%) | 1 (5%) | 0 |
| Neutropenia | 2 (10%) | 5 (25%) | 3 (15%) | 2 (10%) |
| Thrombocytopenia | 2 (10%) | 1 (5%) | 2 (10%) | 1 (5%) |
| Infection | 0 | 2 (10%) | 0 | 2 (10%) |
| Neuropathy | 7 (35%) | 1 (5%) | 0 | 0 |
| Cardiac toxicity | 0 | 0 | 0 | 0 |
| Alopecia | 2 (10%) | 3 (15%) | 0 | 0 |
| Myalgia | 0 | 1 (5%) | 0 | 0 |
| Allergy | 2 (10%) | 1 (5%) | 1 (5%) | 1 (5%) |

[a]ECOG toxicity criteria.

© Cancer Research Campaign 1998

**Figure 1** Overall survival curve of the 20 HCC patients treated with paclitaxel of 0.1 and β of 0.1. Survival estimations were performed according to the Kaplan–Meier method (Kaplan and Meier, 1958). Paclitaxel pharmacokinetic parameters were compared using the Mann–Whitney *U*-test. Treatment-related deaths were compared using Fisher's exact test (one-sided).

### RESULTS

Patient characteristics are listed in Table 1. Twenty patients were studied between November 1993 and September 1994. All patients had AJCC (American Joint Committee on Cancer) stage III or IV HCC (Beahrs et al, 1992). The median age was 64 years. All patients had cirrhosis and elavation of AST but normal bilirubin. Seven patients (35%) had hypoalbuminaemia. Six patients (30%) had prolonged prothrombin time. The median serum AST level was 96 IU $l^{-1}$ (range 46–224; normal 5–45 IU $l^{-1}$). The median serum albumin level was 3.7 g $dl^{-1}$ (range 3.0–4.3; normal 3.7–5.3 g $dl^{-1}$). The median prothrombin time level was 1.22 international normalized ratio (range 1.06–1.40; normal < 1.25). The median total bilirubin level was 0.9 mg $dl^{-1}$ (range 0.5–1.6; normal 0.2–1.6 mg $dl^{-1}$). The median ICG retention ratio was 11% (range 4–51%; normal < 10%) at 15 min. Four patients (20%) had ICG retention ratio ≥ 20%. Cirrhosis was related to chronic HBV and/or hepatitis C virus infection in 80% of patients. Two patients did not return for repeat tumour measurement after one course of paclitaxel treatment. They felt well after treatment and went to China to visit their respective families and did not return to Taiwan. Two patients died of grade 4 neutropenic septicaemia 9 and 16 days after the first cycle of paclitaxel treatment. Sixteen

**Table 3** Individual non-compartmental pharmacokinetic parameters

| Patient number | Dose ($mg\ m^{-2}$) | Cycle number | $C_{max}$ ($\mu g\ ml^{-1}$) | $AUC_{(0-\infty)}$ ($\mu g\ h\ ml^{-1}$) | $t_{1/2}$ (hour) | CLT ($l\ h^{-1}\ m^{-2}$) | $V_{ss}$ ($l\ m^{-2}$) | Per cent urinary recovery | ICG (%) |
|---|---|---|---|---|---|---|---|---|---|
| 1 | 175 | 1 | 5.75 | 20.67 | 18.7 | 8.60 | 55.1 | 3.85 | 3 |
| 2 | 175 | 1 | 4.27 | 24.30 | 11.6 | 7.38 | 51.7 | 9.82 | 5 |
| | 200 | 2 | 6.71 | 34.78 | 11.6 | 5.77 | 39.9 | 2.61 | |
| 3 | 175 | 1 | 4.78 | 21.50 | 8.3 | 8.21 | 46.9 | 12.15 | 11 |
| | 175 | 2 | 3.67 | 13.72 | 9.9 | 16.86 | 83.00 | 5.89 | |
| | 200 | 3 | 6.09 | 21.96 | 12.4 | 9.11 | 56.34 | 10.61 | |
| 4 | 175 | 1 | 5.60 | 24.15 | 14.2 | 7.25 | 49.4 | 9.96 | 20 |
| | 200 | 2 | 5.81 | 30.36 | 13.6 | 6.59 | 47.80 | 7.75 | |
| | 200 | 3 | 9.60 | 40.52 | 14.3 | 4.86 | 34.70 | 5.89 | |
| 5 | 175 | 1 | 4.42 | 23.45 | 10.9 | 7.53 | 50.6 | 6.76 | 13 |
| 6 | 175 | 1 | 3.54 | 16.56 | 9.5 | 10.57 | 55.7 | 5.28 | 4 |
| | 175 | 2 | 3.22 | 10.76 | 6.9 | 16.26 | 80.3 | 3.92 | |
| 7 | 175 | 1 | 4.80 | 22.56 | 13.6 | 7.69 | 73.9 | 9.93 | 31 |
| 8 | 175 | 1 | 4.14 | 20.67 | 10.4 | 8.16 | 56.2 | 4.35 | 17 |
| | 200 | 2 | 3.89 | 21.92 | 11.5 | 9.12 | 72.2 | 12.24 | |
| 9 | 175 | 1 | 3.87 | 17.62 | 8.3 | 9.88 | 70.3 | 2.09 | 18 |
| 10 | 175 | 1 | 3.56 | 17.99 | 11.9 | 9.72 | 59.5 | 4.83 | 12 |
| 11 | 175 | 1 | 4.48 | 19.16 | 5.3 | 9.12 | 40.1 | 3.60 | 5 |
| | 200 | 2 | 5.66 | 24.46 | 10.6 | 8.13 | 51.3 | 0.65 | |
| 12 | 175 | 1 | 4.19 | 33.12 | 9.8 | 5.29 | 56.9 | 11.44 | 23 |
| 13 | 175 | 1 | 3.94 | 16.79 | 12.2 | 10.14 | 141.2 | 5.44 | 14 |
| Mean | 175 | 1 | 4.41 | 21.43 | 11.1 | 8.43 | 62.1 | 6.88 | |
| ± SD | *n* = 13 | | 0.69 | 4.43 | 3.3 | 1.46 | 25.4 | 3.34 | |
| Mean | 175 | 2 | 3.45 | 12.24 | 8.4 | 14.56 | 81.7 | 4.91 | |
| | *n* = 2 | | | | | | | | |
| Mean | 200 | 2 | 5.51 | 27.88 | 11.8 | 7.40 | 52.8 | 5.81 | |
| ±SD | *n* = 4 | | 1.18 | 5.80 | 1.2 | 1.51 | 13.8 | 5.23 | |
| Mean | 200 | 3 | 7.84 | 31.24 | 13.4 | 6.98 | 45.5 | 8.25 | |
| | *n* = 2 | | | | | | | | |

 © Cancer Research Campaign 1998

patients were evaluable for response. The median number of courses of paclitaxel chemotherapy given was two (range 1–7).

There was no complete response (CR) or partial response (PR). There were five stable disease (SD) and 11 progressive disease (PD). The overall survival of patients after paclitaxel treatment is shown in Figure 1. The median survival was 12 weeks (range 1–36). All patients died within 36 weeks after paclitaxel therapy. The median survival of SD patients (14 weeks; range 8–36) was not significantly different from the PD patients (12 weeks; range 8–24).

The toxicities in 20 patients after paclitaxel therapy are listed in Table 2. The major toxicities (grade 3–4) were neutropenia 25%, thrombocytopenia 15%, infection 10%, allergy 10%, diarrhoea 5%, vomiting 5% and mucositis 5%. Two patients (10%) with grade 4 neutropenia died of neutropenic septicaemia 9 and 16 days after paclitaxel treatment. Other toxicities were mild and tolerable.

The paclitaxel pharmacokinetic results are summarized in Table 3. A total of 13 patients were sampled for pharmacokinetics during the first course of paclitaxel at a dose of 175 mg $m^{-2}$. Six of these patients underwent further pharmacokinetic sampling during their second cycle of treatment (two at a dose of 175 mg $m^{-2}$ and four at a dose of 200 mg $m^{-2}$) and two patients were sampled for pharmacokinetics during their third cycle of paclitaxel treatment at a dose of 200 mg $m^{-2}$. For the first cycle of paclitaxel treatment in the 13 patients who were studied for pharmacokinetics at a dose of 175 mg $m^{-2}$, the mean $C_{max}$ was 4.41 ± 0.69 μg $ml^{-1}$ $AUC_{(0-\infty)}$ was 21.42 ± 4.43 (μg h $ml^{-1}$); $t_{1/2}$ was 11.1 ± 3.3 h; CLT was 8.43 ± 1.46 l $h^{-1}$ $m^{-2}$ and $V_{ss}$ was 62.1 ± 25.4 l $m^{-2}$.

In 10 patients whose baseline ICG retention ratio was < 20% at 15 min, the mean paclitaxel $AUC_{(0-\infty)}$ was 19.9 ± 2.7 μg h $ml^{-1}$ and CLT was 8.9 ± 1.1 l $h^{-1}$ $m^{-2}$. In three patients whose baseline ICG retention ratio was ≥ 20% at 15 min, the mean paclitaxel $AUC_{(0-\infty)}$ was 26.6 ± 5.7 μg h $ml^{-1}$ and CLT was 6.7 ± 1.3 l $h^{-1}$ $m^{-2}$ (Table 4) The increase in paclitaxel $AUC_{(0-\infty)}$ ($P < 0.02$) and decrease in CLT ($P < 0.02$) in patients with ICG retention ratio ≥ 20% at 15 min were statistically significant.

The paclitaxel toxicity according to ICG retention ratio < or ≥ 20% at 15 min is shown in Table 5. Grade 3–4 infection and treatment-related deaths were associated with an ICG ratio ≥ 20%. Paclitaxel treatment-related deaths occurred in two out of four (50%) patients with an ICG retention ratio ≥ 20% at 15 min and no deaths occurred in 16 patients with an ICG retention ratio < 20% at 15 min ($P = 0.03$). One of the treatment-related deaths, patient number 12, had pharmacokinetic sampling and had the highest paclitaxel $AUC_{(0-\infty)}$ and the lowest CLT in this study (Table 3).

There were no significant differences in paclitaxel pharmacokinetic parameters in the 13 patients when baseline AST value above or below two times normal, presence or absence of ascites, AFP above or below 400 ng $ml^{-1}$, albumin level above or below 3.5 g $dl^{-1}$ or prothrombin time above or below 1.25 times control were compared.

### DISCUSSION

The results of this study indicate that paclitaxel may have no significant anti-cancer activity against HCC. Despite the excellent anti-cancer effects of paclitaxel in the treatment of various cancers, no response was observed in HCC patients after paclitaxel chemotherapy of 175–200 mg $m^{-2}$ over 3 h in this study. The median survival of HCC patients after paclitaxel chemotherapy in this study was 12 weeks and the absence of survivors after 36 weeks suggest that paclitaxel may have no major clinical impact on the survival of HCC patients. These results appear similar to median survival of 13 weeks in HCC patients treated by ineffective chemotherapy reported by Lai et al (1989). Investigation of other new effective chemotherapeutic agents for the treatment of HCC is urgently needed.

The mechanism of drug resistance of HCC to paclitaxel is uncertain. One possibility may be the intrinsic high expression of the multiple drug resistance (*mdr*) gene in human hepatic tissue and hepatoma (Goldstein et al, 1989). Paclitaxel resistance in cancer patients may be associated with high expression of the *mdr* gene (Horwitz et al, 1993; Webster et al, 1993).

The incidence of 25% grade 3–4 neutropenia in this study is similar to the 27–29% reported when paclitaxel was given in the same dose and schedule to treat ovarian cancer patients with normal liver function (Rowinsky et al, 1993*b*; Guastalla et al, 1994). Overall toxicities of paclitaxel in this study also appear similar to other paclitaxel studies, but the 10% treatment-related deaths in this study appeared higher than reported (Rowinsky et al, 1993*b*; Chan et al, 1994; Gianni et al, 1994; Guastalla et al, 1994).

As paclitaxel is metabolized by the cytochrome P-450 system (Jamis-Dow et al, 1995), patients with impaired liver function may be at increased risk of paclitaxel toxicities. Indeed, Wilson et al (1994) reported that five patients with metastatic liver disease and alanine aminotransferase > 2 times normal had significantly decreased paclitaxel clearance and increased paclitaxel toxicity when paclitaxel was given by a 96-h infusion at doses ranging from 120 to 160 mg $m^{-2}$. Venook et al (1994*b*) reported significant paclitaxel

**Table 4** Paclitaxel pharmacokinetic parameters and indocyanine green (ICG) test in 13 patients

| | ICG < 20% ($n$ = 10) | ICG ≥ 20% ($n$ = 3) |
|---|---|---|
| $C_{max}$ (μg $ml^{-1}$) | 4.27 ± 0.65 | 4.86 ± 0.70 |
| $AUC_{(0-\infty)}$ (μg h $ml^{-1}$) | 19.9 ± 5.7[a] | 26.6 ± 5.7[a] |
| $T_{1/2}$ (hr) | 10.7 ± 3.5 | 12.5 ± 2.4 |
| CLT (l $h^{-1}$ $m^{-2}$) | 8.9 ± 1.1[a] | 6.7 ± 1.3[a] |
| $V_{ss}$ (l $m^{-2}$) | 62.7 ± 28.7 | 60.1 ± 12.6 |
| Per cent urinary recovery | 5.81 ± 3.0 | 10.4 ± 0.8 |

[a]$P < 0.02$.

**Table 5** Paclitaxel toxicity according indocyanine green (ICG) retention ratio in 20 patients

| Toxicities (≥ grade 3) | ICG < 20% ($n$ = 16) | ICG ≥ 20% ($n$ = 4) |
|---|---|---|
| Neutropenia | 2 (12.5%) | 3 (75%) |
| Thrombocytopenia | 1 (6.25%) | 2 (50%) |
| Infection | 0 | 2 (50%)[a] |
| Treatment-related deaths | 0 | 2 (50%)[a] |
| Vomiting | 1 (6.25%) | 0 |
| Diarrhoea | 0 | 1 (25%) |
| Mucositis | 0 | 1 (25%) |
| Allergy | 1 (6.25%) | 1 (25%) |

[a]$P = 0.03$ (Fisher's exact test, one-sided).

© Cancer Research Campaign 1998

toxicities in cancer patients with AST > 2 times normal with or without hyperbilirubinaemia receiving paclitaxel as a 24-h infusion and recommended reduction in the paclitaxel dose in these patients. No treatment-related deaths were mentioned in these two abstracts.

This is the first report of the relationship between paclitaxel toxicity, pharmacokinetics and liver impairment when paclitaxel was given as a 3-h infusion. All patients in this study had normal bilirubin but extensive replacement of the liver by HCC and cirrhosis. Most patients had some hepatic impairment, as indicated by abnormal transaminase, albumin or prothrombin time. These liver function tests were of no predictive value in paclitaxel clinical toxicity or altered paclitaxel pharmacokinetic in this study. Accurate prediction of severe paclitaxel toxicity in patients with liver impairment is clinically important. A more sensitive or predictive test will be useful, especially in patients with normal bilirubin but subclinical impairment of liver function.

The ICG retention test is a simple and accurate liver function test to determine liver function as well as hepatic blood flow (Cherrick et al, 1960; Caesor et al, 1961). The ICG retention test has a good prognostic value in cirrhotic patients and is widely accepted for measuring functional reserve in chronic liver diseases. The ICG retention test is also useful in assessing hepatic functional reserve preoperatively to predict successful hepatic resection in HCC patients (Okamoto et al, 1984). In our hospital, the ICG retention ratio < 20% at 15 min has been used for the last 8 years to select HCC patients with adequate hepatic functional reserve for hepatic surgery (Jwo et al, 1992; Wu et al, 1996). The ICG retention ratio ≥ 20% at 15 min may be predictive of increased paclitaxel toxicities including treatment-related deaths associated with liver impairment and increased AUC and reduced clearance of the drug.

These findings may be important clinically because paclitaxel is usually given at full dose to patients with normal bilirubin and some patients may have significant toxicity. We recommend that paclitaxel dose reduction should be considered in patients with normal bilirubin but subclinical hepatic impairment. High-risk patients with extensive replacement of liver by tumour, cirrhosis, chronic HBV infection, etc. may be identified by an additional liver function test such as the ICG retention test. Further clinical investigation of paclitaxel pharmacokinetics and liver function may be worthwhile.

In conclusion, paclitaxel had no significant anti-cancer activity in patients with advanced hepatocellular carcinoma when administered at a dose of 175–200 mg $m^{-2}$ using a 3 h intravenous infusion. ICG retention ≥ 20% at 15 min appeared to identify patients with normal bilirubin and subclinical liver impairment and to predict altered paclitaxel pharmacokinetics and increased paclitaxel clinical toxicities, including treatment-related deaths. Paclitaxel should be used with caution in patients with hepatic dysfunction. Further clinical investigation of paclitaxel pharmacokinetics in patients with liver impairment is warranted.

### ACKNOWLEDGEMENTS

This study is partially supported by a grant from the Department of Health, Executive Yuan, Taiwan, Republic of China

### REFERENCES

Beahrs OH, Henson DE, Hutter RVP and Kennedy BJ (eds) (1992) *Manual for Staging of Cancer. American Joint Committee on Cancer*. 4th edn. Lippincott: Philadelphia

Caesar J, Shaldon S and Chiandussi L (1961) The use of indocyanine green in the measurement of hepatic blood flow and as a test of hepatic functions. *Clin Sci* **21**: 43–57

Chan WK, Lin TH, Liu M, Chen YM, Wu MF and Whang-Peng J (1994) A pilot study of taxol treatment in carcinoma of unknown primary site: preliminary results. *Therapeut Radiol Oncol* **3**: 217–227

Cherrick GR, Stein SW, Leevy CM and Davidson CS (1960) Indocyanine green: observation on its physical properties, plasma decay and hepatic extraction. *J Clin Invest* **39**: 592–600

Farmen RH, Muniak JF and Pittman KA (1987) Management of pharmacokinetic data using HP-3357/mainframe IBM interfacing. *Drug Information J* **21**: 141–152

Gianni L, Capri G, Munzone E and Straneo M (1994) Paclitaxel (taxol) efficacy in patients with advanced breast cancer resistant to anthracyclines. *Semin Oncol* **21**(S8): 29–33

Goldstein LJ, Galski H, Fojo A, Willingham M, Lai SL, Gazdar A, Pirker R, Green A, Crist W and Brodeur GM (1989) Expression of a multiple drug resistance gene in human cancers. *J Natl Cancer Inst* **81**: 116–124

Guastalla JP, Lhomme C, Dauplat J, Namer M, Bonneterre J, Oberling F, Pouillart P, Fumoleau P, Kerbrat P and Tubiana N (1994) Taxol (paclitaxel) safety in patients with platinum pretreated ovarian carcinoma: an interim analysis of a phase II multicenter study. *Ann Oncol* **5**(S6): S33–38

Guchelaar HJ, ten Napel CH, de Vries EG and Mulder NH (1994) Clinical, toxicological and pharmaceutical aspects of the antineoplastic drug taxol: a review. *Clin Oncol* **6**: 40–48

Horwitz SB, Cohen D, Rao S, Ringel I, Shen HJ and Yang CP (1993) Taxol: mechanisms of action and resistance. *M Natl Cancer Inst* **15**: 55–61

Jamis-Dow CA, Klecker RW, Katki AG and Collins JM (1995) Metabolism of taxol by human and rat liver in vitro: a screen for drug interactions and interspecies differences. *Cancer Chemother Pharmacol* **6**: 107–114

Jwo SC, Chiu JH, Vhan GGY, Loong CC and Lui WY (1992) Risk factors linked to tumor recurrence of human hepatocellular carcinoma after hepatic resection. *Hepatology* **16**: 1367–1371

Kaplan EM and Meier P (1958) Nonparametric estimation from incomplete observations. *J Am Statis Assoc* **53**: 457–481

Lai KH, Tsai YT, Lee SD, Ng WW, Teng HC, Tam TN, Lo GH, Lin HC, Lin HJ, Wu JC, Lay CS, Wang SS and Chan WK (1989) Phase II study of mitoxantrone in unresectable primary hepatocellular carcinoma following hepatitis B infection. *Cancer Chemother Pharmacol* **23**: 54–56

Muir C (1989) *Cancer Incidence in Five Continents*. IARC: Lyons France

Okamoto E, Kyo A, Yamanaka N, Tanaka N and Kuwata K (1984) Prediction of the safe limit of hepatectomy by combined volumetric and functional measurements in patients with impaired hepatic function. *Surgery* **95**: 586–592

Oken MM, Creech RH, Tormey DC, Horton J, Davis TE, McFadden ET and Carbone PP (1982) Toxicity and response criteria of the Eastern Cooperative Oncology Group. *Am J Clin Oncol* **5**: 649–655

Riegelman S and Collier P (1984) An application of statistical moment theory to the evaluation of in vivo dissolution time and absorption time. *J Pharmaco Biopharm* **8**: 509–534

Rowinsky EK (1994) Update on the antitumor activity of paclitaxel in clinical trials. *Ann Pharmaco* **28**(S): S18–22

Rowinsky EK, Wright M, Monsarrat B, Lesser GJ and Donehower RC (1993*a*) Taxol: pharmacology, metabolism and clinical implications. *Cancer Surv* **17**: 283–304

Rowinsky EK, Eisenhauer EA, Chaudhry V, Arbuck SG and Donehower RS (1993*b*) Clinical toxicities encountered with paclitaxel (taxol). *Semin Oncol* **20**(S3): 1–15

Simon R (1989) Optimal two-stage designs for phase II clinical trials. *Control Clin Trials* **10**: 1–10

Sonnichsen DS, Hurwitz CA, Pratt CB, Shuster JJ and Relling MV (1994) Saturable pharmacokinetics and paclitaxel pharmacodynamics in children with solid tumors. *J Clin Oncol* **12**: 532–538

Tong MJ, Sun SC, Schaeffer BT, Chang NK, Lo KJ and Peters RL (1971) Hepatitis-associated antigen and hepatocellular carcinoma in Taiwan. *Ann Intern Med* **75**: 687–691

Venook AP (1994*a*) Treatment of hepatocellular carcinoma: too many options? *J Clin Oncol* **12**: 1323–1334

Venook AP, Egorin M, Brown TD, Batist G, Budman DR, Rosner GL, Jahan TM and Schilsky RL (1994*b*) Paclitaxel (taxol) in patients with liver dysfunction. Proc *Am Soc Clin Oncol* **13**: A350

Webster L, Linsenmeyer M, Millward M, Morton C, Bishop J and Woodcock D (1993) Measurement of cremophor EL following taxol: plasma level sufficient to reverse drug exclusion mediated by the multidrug-resistant phenotype. *J Natl Cancer Inst* **85**: 1685–1690

© Cancer Research Campaign 1998

Willey TA, Bekos EJ, Gaver RC, Duncan GF, Tay LK, Beijnen JH and Farman RH (1993) High-performance liquid chromatographic procedure for the quantitative determination of paclitaxel (Taxol) in human plasma. *J Chromatogr* **621**: 231–238

Wilson WH, Berg SL, Bryant G, Wittes RE, Butes S, Fojo A, Steinberg SM, Goldspiel BR, Herdt J and O'Shaughnessy J (1994) Paclitaxel in doxorubicin-refractory or mitoxantrone-refractory breast cancer: a phase I/II trial of 96-hour infusion. *J Clin Oncol* **12**: 1621–1629

Wu CC, Ho WL, Yeh DC, Huang CR, Liu TJ and P'eng FK (1996) Hepatic resection of hepatocellular carcinoma in cirrhotic liver: is it unjustified in impaired liver function? *Surgery* **120**: 34–39

© Cancer Research Campaign 1998